# Concepts for a therapeutic prolongation of nephrogenesis in preterm and low-birth-weight babies must correspond to structural-functional properties in the nephrogenic zone

**DOI:** 10.1186/s40348-017-0078-6

**Published:** 2017-12-07

**Authors:** Will W. Minuth

**Affiliations:** Institute of Anatomy, University of Regensburg, 93053 Regensburg, Germany

## Abstract

Numerous investigations are dealing with anlage of the mammalian kidney and primary development of nephrons. However, only few information is available about the last steps in kidney development leading at birth to a downregulation of morphogen activity in the nephrogenic zone and to a loss of stem cell niches aligned beyond the organ capsule. Surprisingly, these natural changes in the developmental program display similarities to processes occurring in the kidneys of preterm and low-birth-weight babies. Although those babies are born at a time with a principally intact nephrogenic zone and active niches, a high proportion of them suffers on impairment of nephrogenesis resulting in oligonephropathy, formation of atypical glomeruli, and immaturity of parenchyma. The setting points out that up to date not identified noxae in the nephrogenic zone hamper primary steps of parenchyma development. In this situation, a possible therapeutic aim is to prolong nephrogenesis by medications. However, actual data provide information that administration of drugs is problematic due to an unexpectedly complex microanatomy of the nephrogenic zone, in niches so far not considered textured extracellular matrix and peculiar contacts between mesenchymal cell projections and epithelial stem cells via tunneling nanotubes. Thus, it remains to be figured out whether disturbance of morphogen signaling altered synthesis of extracellular matrix, disturbed cell-to-cell contacts, or modified interstitial fluid impair nephrogenic activity. Due to most unanswered questions, search for eligible drugs prolonging nephrogenesis and their reliable administration is a special challenge for the future.

## Introduction

The physiological adaption of a newborn baby to extrauterine life depends on many parameters including an intact morphological and functional development of the kidneys before and after birth [1]. Usually the complex process of nephron induction is completed at the time of birth. However, when a baby is born preterm or with low birth weight, the kidneys are still in a process of active nephrogenesis [2–4]. Autopsied preterm kidneys and a baboon model of preterm birth reveal that nephrogenesis is able to proceed for up to 3 weeks in extrauterine life [5].

Now, there is more and more evidence that preterm delivery is interfering the process of nephrogenesis resulting in oligonephropathy, which is estimated to be between 8 and 24% [3, 4]. Pleiotropic noxae such as unbalanced metabolites, misleading morphogen signaling, inflammation, hyperoxigenation, low-protein diet, hypoperfusion, or nephrotoxic drugs are held accountable to cause it. Related pathologic data further exhibit that in neonatal kidneys, up to 18% morphologically abnormal glomeruli are present that illustrate a dilated Bowman’s space and a shrunken glomerular tuft [6]. Most importantly, occurrence of such glomeruli is restricted to the outer renal cortex pointing out that the nephrogenic zone, involved stem cell niches, and the last nephron generation are affected.

A possible therapeutic aim for preterm and low-birth-weight babies is to compensate impairment of nephrogenesis by medication and to stimulate morphogenic activity in the nephrogenic zone to prolong thereby nephrogenesis during early postnatal development [7]. However, all of the presently available data point out that such an approach is difficult to perform, since it must be adapted to peculiar structural and functional features contained in the nephrogenic zone [8]. Before doing so, there is also an urgent necessity of investigations dealing in human fetal kidney with the synthesis, secretion, and concrete transport of morphogens locally involved in maintenance of stemness and nephrogenesis [9]. Finally, since data for the application of drugs via the local blood supply and distribution in this peculiar environment is missing, a promising concept for prolongation of nephrogenesis is awaiting solid implementation.

## Review

### View onto the nephrogenic zone

**Fig. 1:**
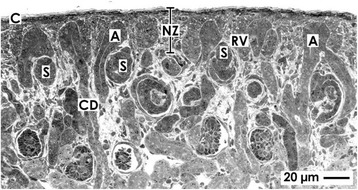
Optical microscopy of the nephrogenic zone (NZ) of a 1-day-old glutaraldehyde-fixed rabbit kidney. The section lines perpendicular to the organ capsule (C) and in parallel to the lining collecting duct (CD) tubules, which form at their endings a CD ampulla (A). At the lateral aspect of a CD ampulla renal vesicles (RV) or S-shaped bodies (S) indicate active nephrogenesis

During fetal growth of a mammalian kidney, the nephrogenic zone is restricted to the cortex corticis of parenchyma [10]. A factual problem is that randomly cut sections do not help to recognize essential structural details in microscopic analysis. Hence, to obtain comparable perspectives, a monopapillary kidney of a mouse, rat, or rabbit is divided in the middle between both poles for histological preparations. A human fetal kidney is cut best from the capsule towards the papilla of a lobus. Following this advice, the section plane shows the parenchyma in the cortices that is orientated along the lumen of the lining collecting duct (CD) tubules and perpendicular to the capsule (Fig. 1). Due to incomplete histological preservation, pathological specimens of a human kidney are often hard to interpret. To overcome some of the barriers, the nephrogenic zone of a neonatal rabbit fixed under controlled conditions shall serve here for illustration.

### Eccentric position

The nephrogenic zone extends as a band along the entire surface of a fetal kidney and shows a width between 60 and 100 μm depending on species [11]. Sections stained by hematoxylin-eosin solution present the nephrogenic zone as a “blue strip” [12]. Its outer aspect is covered by the organ capsule, while the inner side is facing maturing CD tubules and developing nephrons including first stages of arising glomeruli (Fig. 1). Located between these two limits, the complete cell biological machinery is contained here to maintain cell stemness, induction, and initial development of nephrons. Concrete tissues of the nephrogenic zone are the ureteric bud-derived collecting duct (CD) tubules, related branching sites including CD ampullae, the mesenchyme, and the capsule.

### Compartments

**Fig. 2:**
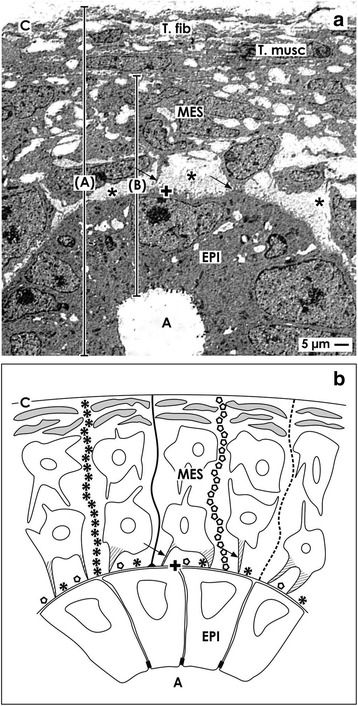
Occurrence of stem cells in a 1-day-old and glutaraldehyde-fixed rabbit kidney shown by **a** transmission electron microscopy and **b** schema. **a** The capsule (C) consists of a tunica fibrosa (T.fib) and muscularis (T.musc). Epithelial (EPI) stem cells are enclosed in the tip of a CD ampulla (A) covered by a basal lamina (cross). An interface (asterisks) separates mesenchymal (MES) from epithelial stem cells. Projections of mesenchymal cells (arrow) cross it to contact a CD ampulla. Both the nephrogenic zone and the capsule can be seen as a niche that is covering the entire organ (A). In contrast, only the tip of a CD ampulla containing epithelial stem cells and some above positioned GDNF^+^/Six2^+^/CITED1^+^ mesenchymal cells are regarded as a single niche (B). **b** Histochemistry exhibits that in the basal lamina of a CD ampulla tip, laminin ɣ1 and agrin are contained. Further microfibers binding soybean agglutinin (SBA; black line) and anti-collagen type I (black asterisks), type II (light circles), and type III (dotted line) originate here to cross the interface and mesenchymal stem cells for fastening the capsule [10]

As illustrated by low magnification, the outer border of the nephrogenic zone is the organ capsule (Fig. 1 and 2a). In homology to human, in neonatal rabbit kidney, it consists of a tunica fibrosa and a tunica muscularis [10]. Beyond the capsule, only two layers of metanephric mesenchymal (MES) stem cells occur. Beneath them and separated by a striking interface, CD ampullae are aligned. All of them have the same orientation. Their tips have a distance of 14 μm to the inner side of the organ capsule [13]. Further down and directed towards maturing nephrons, the dilated part of a CD ampulla continues to a neck and then to a shaft, which is connected with a differentiating CD tubule [14]. This site reflects the inner limit of the nephrogenic zone. Depending on the state of nephron formation at the upper later aspect of a CD ampulla, either mesenchymal cell condensation in the form of pretubular aggregates, polarized renal vesicles, or comma- or S-shaped bodies occur.

### Stem cell niches

The capsule and the nephrogenic zone contain different kinds of stem cells (Fig. 2a). Mesenchymal stem cells occur as well in the capsule as in the underlying metanephric mesenchyme [15, 16]. Epithelial stem cells are contained in the tip of a CD ampulla [17]. Since both parenchyma and stroma are developing from this pool, the capsule, mesenchymal as epithelial stem cells within the nephrogenic zone represent an extended stem cell niche that is covering the entire fetal kidney. In contrast, in a different definition, only the tip of a CD ampulla with contained epithelial stem/progenitor cells and some facing nephrogenic (GDNF^+^/Six2^+^/CITED1^+^) mesenchymal cells are regarded as an individual niche [18–20]. It is obvious that only this latter definition points to the site, where induction and initial formation of an individual nephron take place.

### Exact coordination during branching

Development of renal parenchyma proceeds by a process, which is defined as branching morphogenesis [21–23]. In a human kidney, the invading ureteric bud produces first the pelvis and calyceal sytems in the presumptive medulla. After formation of the ducts of Bellini, successive elongation of CD tubules in the cortex takes place. Surprisingly, during radial extension of parenchyma, a change of the branching pattern in the inner and then in the outer cortex takes place [24]. Before birth, in the nephrogenic zone of a human and rabbit kidney, this spatiotemporal program raises in elongating CD tubules bifid branches. Their endings are orientated towards the covering mesenchyme. Since a branch exhibits a dilated form, it was specified as a CD ampulla. Actual investigations show that the process of CD tubule branching depends as well on the transcription factor Foxd1 as on the renin-angiotensin system (RAS) [25]. Related survival, proliferation, and primary differentiation of branching CD cells are influenced by fibroblast growth factors (FGFs) and retinoic acid (RA) [26].

For the human kidney, concrete data are lacking, but in the neonatal rabbit kidney, approaching of epithelial and mesenchymal stem cells is prescribed by microfibers that link the inner side of the organ capsule with the tip of the CD ampullae (Fig. 2b). In its basal lamina, laminin ɣ1 and the proteoglycan agrin are contained. At this site also microfibers labeled by anti-collagen types I, II, III respectively soybean agglutinin (SBA) originate [10]. They span through the two layers of nephrogenic mesenchymal stem cells for mounting on the inner side of the capsule. This mechanic construction demonstrates that epithelial and mesenchymal stem cells do not meet by accident but are integral part of a guiding cage. It coordinates positioning at a distinct site and keeps contained cells close to the capsule.

Exact movement of involved cells within an individual niche is adjusted by the secreted bone morphogenic protein (BMP) antagonist Cerberus homologue1 (Cer1), Ret protooncogene (Ret), and ETS translocation variant4 (Etv4) [17, 27]. By molecular positioning the tip of a CD ampulla, the inner layer of the nephrogenic mesenchymal cells comes in close vicinity to the epithelial stem cells. While approaching, some of the mesenchymal cells acquire competence so that they can respond to morphogens. This operation is directed by protocadherin (cadherin family member14: FAT4/dachsous cadherin related1: Dchs1) signaling [28]. Yet, epithelial stem cells in the tip of a CD ampulla are exactly vis-à-vis of GDNF^+^/Six2^+^/CITED1^+^ mesenchymal cells [19, 20]. When the exchange of morphogens was successful, only the group of induced mesenchymal cells separates, aggregates to become a pretubular aggregate, and performs a mesenchyme-to-epithelial transition (MET) to develop into a polarized renal vesicle at the lateral aspect of the related CD ampulla. For the remaining cells in the mesenchyme, the transcription factor Zeb1 is controlling the proceeding balance between proliferation, migration, and apoptosis [29].

When impairment of nephrogenesis in the kidney of preterm infants is under debate, remodeling of illustrated microfibers appears to be an important issue (Fig. 2b) [30]. This process requires tissue transglutaminases (TGases), matrix metalloproteinases (MMPs) and membrane targeted MMPs (MT-MMPs) controlling synthesis, and degradation of extracellular matrix [31, 32]. An equilibrated activity of those TGases directs also growth factor-stimulated signaling and in turn cell proliferation [33, 34]. Whether the balance of TGase activity on microfibers or on the basal lamina of a CD ampulla tip is disturbed and intrinsic activity within renal stem cell niches of preterm infants is impaired thereby, waits for investigation. However, when an increased activity is detected, therapeutic application of inhibitors of TGases may help to find back to an intact equilibrium between synthesis and degradation [35].

### Separation of stem cells by an interface

**Fig. 3:**
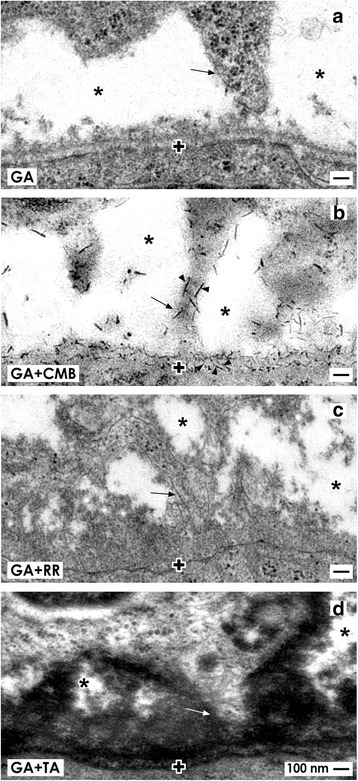
Transmission electron microscopy of a 1-day-old and glutaraldehyde-fixed rabbit kidney depicts mesenchymal cell projections (arrow) contacting epithelial cells and unveils extracellular matrix at the interface of the renal niche. **a** Specimens fixed by conventional glutaraldehyde (GA) solution illustrate a clear interface (asterisk). **b** In contrast, fixation by glutaraldehyde solution including cupromeronic blue (CMB) shows that numerous braces of proteoglycans are recognized on the surface of cell projections and within the basal lamina (arrow head) of epithelial stem cells. **c** Specimens fixed by GA solution including ruthenium red (RR) or **d** tannic acid (TA) unmasks further textured extracellular matrix at the interface and basal lamina of a CD ampulla, which is labeled by a cross

For induction of a nephron, epithelial and mesenchymal cell bodies within the niche are approaching but stand at a distance between 1 to 2 μm (Figs. 2a and 3a) [36, 37]. This special configuration was recorded as well by optical microscopy [38–41] as transmission electron microscopy (Fig. 2a and 3) [42–45]. Although less considered, it is present in mouse, rat, rabbit, and human kidneys. Moreover, ultrastructural analysis exhibits that a basal lamina covers the tip of a CD ampulla. Its lamina fibroreticularis consists of a remarkable fibrillar meshwork [46]. Further projections (also called cytonemes, signaling filopodia, protrusions) of mesenchymal cells cross the interface to penetrate the basal lamina and to contact the basal plasma membrane of epithelial stem cells (Figs. 2a and 3a). Surprisingly, the interface looks blank, when conventional fixation by glutaraldehyde (GA) solution for transmission electron microscopy is used. However, numerous braces of proteoglycans on the surface of projections and on the basal lamina become visible, when fixation of specimens is performed by GA solution including cupromeronic blue (Fig. 3b) [47]. Application of GA solution including ruthenium red (Fig. 3c) or tannic acid (Fig. 3d) unveils earlier unseen textured extracellular matrix [37]. Comparable to before mentioned microfibers, one has to imagine that any disparity in synthesis or degradation of illustrated extracellular matrix at the interface will impair nephrogenesis due to disturbed binding of morphogens as it is described later.

### Contacts between cells

Although the bodies of mesenchymal and epithelial stem cells are separated by a striking interface, projections of mesenchymal cells cross it, to establish a contact at the tip of a CD ampulla (Figs. 2a and 3) [48]. Earlier, it was reported that at this site, integrin α8β1 is localized, which binds to nephronectin on the basal lamina covering epithelial stem cells [49–51]. In addition, the micro-tubule-dependent motor protein kinesin (KIF26B) is established here to control cell attraction, signal transduction, and developmental patterning [52–54].

**Fig. 4:**
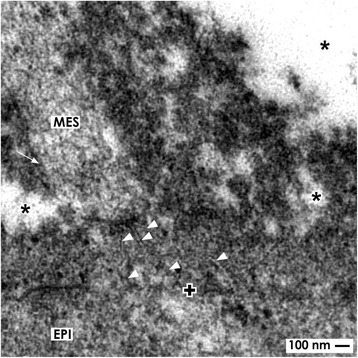
Transmission electron microscopy of a 1-day-old and glutaraldehyde-fixed rabbit kidney shows cell-to-cell contacts in the renal stem cell niche. A projection (arrow) of a mesenchymal cell (MES) is crossing the interface to establish a contact with epithelial (EPI) cells via tunneling nanotubes (arrow head). The basal lamina of epithelial cells on a CD ampulla is marked by a cross

Moreover, new data show that a mesenchymal cell projection penetrates the basal lamina of epithelial stem cells. Its distal portion is in contact with extracellular matrix that creates a special sleeve [48]. Between the end of a mesenchymal cell projection and the basal plasma membrane of an epithelial stem cell, tunneling nanotubes are established (Fig. 4). This essential finding points to a concrete path between mesenchymal and epithelial cells that is optimally suited for cell-to-cell communication including the transport of a variety of molecules [55]. While in transmission electron microscopy, the moment an actual physiological status is frozen, actual timelaps imaging shows that mesenchymal cells are motile so that they attach and detach from the CD ampulla tip across time [56]. One can imagine that any disturbance of anchorage or communication between mesenchymal and epithelial stem cells including restricted functionality in cell projections and/or tunneling nanotubes will impede nephron induction.

### Minimal vascular supply

**Fig. 5:**
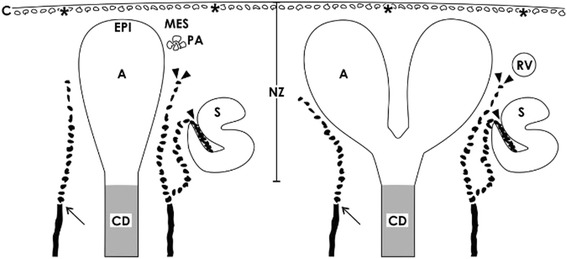
Schematic illustration informs about the incomplete vascular supply of the nephrogenic zone (NZ) in a neonatal rabbit kidney. Label by antibody EC1 [58] depicts that endothelial cells arise from cortical radiate arteries (arrow) lining in parallel to the collecting duct (CD) tubules. Endothelial cells (arrow heads) migrate to the lateral aspect of an ureteric bud derived CD ampulla (A) and to the lower cleft of a S-shaped body (S). In addition, cells of the tunica muscularis within the organ capsule (C) form intracellular and extracellular tunnels (asterisks) for production of interstitial fluid [10]. It is obvious that the area of the niche including the tip of a CD ampulla with epithelial (EPI) cells and neighboring mesenchymal cells (MES) is avascular. PA pretubular aggregate, RV renal vesicle

It is hard to believe, but the incomplete vascular supply is a unique feature of the nephrogenic zone. Earlier performed histochemistry with *Ulex europaeus* I lectin on human fetal kidneys informs that in the area of starting nephrons, not intact capillaries but only strands of spreading endothelial cells exist [57]. In a neonatal rabbit kidney, it was recorded by immunohistochemistry that capillaries line from cortical radiate arteries towards the cortex corticis. Forming vessels are recognized on developing glomeruli. Further strands of endothelial cells line at the lateral aspect of a CD ampulla to the lower cleft of S-shaped bodies, where the glomerular tuft is arising (Fig. 5) [58]. Actual immunohistochemical data with the endothelium marker anti-CD 31 exhibits that cell strands of forming vessels occur at the branching site of the CD tubule and at the lateral aspect of a CD ampulla [59, 60]. However, at the niche site including the tip of a CD ampulla and neighboring mesenchymal stem/progenitor cells, endothelial cells are not present.

Expression of endothelial nitric oxide synthase was detected in the kidney of a rat on developing S-shaped bodies, but not within the mesenchymal cell layers [61]. In homology to the ureteric bud, it appears most probable that Wingless Int-1 (Wnt7b) protein expressed by epithelial stem cells at the lateral aspect of CD ampullae activates canonical Wnt signaling in the surrounding interstitial cells to establish capillaries [62]. An actual investigation on developing mouse kidney exhibits that forming vessels at the nephrogenic zone remain un-perfused, although oxygenation is able to drive nephron progenitor differentiation [63, 64]. Finally, also capillaries within the capsule produce interstitial fluid to transport it in a complex tunnel system towards the outer side of the nephrogenic zone (Fig. 5) [10].

### Integrated signaling of morphogens

Growth factors or more precisely morphogens are highly bioactive molecules, which control stem cells within the nephrogenic zone and individual niches during development of the kidney [65]. This complex task comprises regulation of stemness, cell proliferation, targeted migration, competence, induction, and primary formation of nephrons [9]. In a human kidney, this process is active from the beginning of organ anlage up to birth, while in other mammalian species, nephrogenesis can proceed during the early postnatal period [7].

More general tasks such as supervising of survival, stemness, and proliferation of stem cells in the nephrogenic zone is triggered for example by morphogen mouse double minute 2 homolog (Mdm2). Its successful signaling results in expression of site-specific markers such as amphiphysin, Cited1, Sall1, and Pax2 [66].

A clearly focused role has morphogens, which operate competence, induction, and subsequent nephron formation. It starts when an elongating CD tubule divides into bifid branches to form CD ampullae [67]. To make signaling of a morphogen successful, on neighboring mesenchymal cells, the time slot for competence must open. In that case, morphogen BMP7 makes possible that related mesenchymal stem cells principally can answer an inductive signal [68]. However, during the process of nephron induction, not all GDNF^+^/Six2^+^/CITED1^+^ mesenchymal cells will respond, but only those that are exactly facing epithelial cells contained in the tip of a CD ampulla [19, 20]. Downregulation of Six2^+^ by knockdown of neurofibromin with small interfering RNAs results in loss of competence, lack of nephron induction, and an increased ratio of apoptotic cells [69]. Further repetitive nephron induction requires an adequate pool of competent cells at the right time and place. If reduced, it is refilled by self-renewing of progenitor cells in the nephrogenic zone operated by intact Six2^+^ expression [70, 71].

When a correct number of competent nephrogenic mesenchymal cells in a niche is available, induction of a nephron takes place. It is triggered by complex signaling of locally synthetized morphogens [9, 65]. Since site of their expression and receptors on target cells are in many cases not identical, morphogens are transported between competent mesenchymal and epithelial stem cells contained in the tip of a CD ampulla (Figs. 2, 3, and 4). The main players in this concert are bone morphogenetic proteins (BMP4, BMP7), fibroblast growth factor (FGF8), glial cell line-derived neurotrophic factor (GDNF), Wnt family members (Wnt4, Wnt5a, Wnt9b), related receptors, and involved modulators [9, 65]. A list of them acting in chronical sequence between induction of competent mesenchymal cells and formation of a polarized renal vesicle is presented in Table 1.

**Table 1:** List of selected morphogens acting during the initial phase of nephrogenesis

Morphogen	Function	Expression site	Receptor in target	References
Wnt9b	Induction of nephrogenesis, mesenchymal-to-epithelial transition (MET)	CD ampulla	FZD (Frizzled)/LRP receptor-related protein in mesenchyme	[97]
BMP4	Prevention of ectopic branching of CD tubule	Mesenchyme	Activin-like kinase 3/6 (Alk)/type II receptor in CD ampulla	[131]
BMP7	Branching of CD ampulla, competence and receptivity in mesenchyme	Mesenchyme	Activin-like kinase 3/6 (Alk)/type II receptor in CD ampulla in mesenchyme	[68, 131]
GDNF	Elongation of CD tubule, branching of CD ampulla, site-specific cell proliferation	Mesenchyme	Receptor tyrosine-protein kinase (Ret)/GDNF f-mily receptor Alpha 1 (GFRα1) in CD ampulla	[80]
Wnt5a	Clear position of mesenchyme, induction of nephrogenesis	CD ampulla	FZD (Frizzled)/LRP receptor-related protein In mesenchyme	[132]
Wnt4	Induction of nephrogenesis, mesenchymal-to-epithelial transition (MET)	Condensing mesenchyme Pretubular aggregate	FZD (Frizzled)/LRP receptor-related protein in mesenchyme	[74, 133]
Fgf8	Transition of induced mesenchyme into pretubular aggregate and renal vesicle	Mesenchyme Renal vesicle	Fibroblast growth factor receptor 1 and 2 (Fgfr1/2) in mesenchyme	[98, 103, 134]

When signaling of involved morphogens was successful, a small group of induced GDNF^+^/Six2^+^/CITED1^+^ mesenchymal cells separates and aggregates to perform a mesenchymal-to-epithelial transition (MET) on the upper lateral aspect of a related CD ampulla. Running nephrogenesis is histologically recognized first by signs of cell condensation, formation of a pretubular aggregate, and then development of a renal vesicle [19, 20, 72].

### Morphogens must reach related receptors

Previously, one believed that epithelial and mesenchymal stem cells within the renal niche meet by chance and that transport of morphogens occurs by diffusion. It was further speculated that the distance between interacting cells can be neglected [9, 67]. Under these circumstances, a sharp gradient principally can arise and an effective concentration of a secreted morphogen will reach its related receptor [73]. However, these arguments are pure speculations and were not investigated for the renal niche.

Clear but little noticed hints about the transport of a morphogen provide transfilter culture experiments. For example, NIH3T3 mouse embryo fibroblast cells expressing morphogen Wnt4 were cultured for tubule induction on the one side, while isolated nephrogenic mesenchyme was placed on the other side of a filter [74]. It was demonstrated that separating filters with pore sizes of 0.1 μm and above support induction including tubule formation, while pores of 0.05 μm abolish it. One must consider that morphogens are so small molecules that they can cross a pore of this diameter without any problem. It may surprise but solubilized morphogen molecules in form of a supernatant from Wnt4 expressing cells were not able to induce formation of tubules in isolated nephrogenic mesenchyme. Thus, the transport of a morphogen during induction of a nephron does not work exclusively by simple diffusion but appears to be more complex [75].

Moreover, earlier morphological findings show cell-to-cell contacts between mesenchymal and epithelial stem cells in niches of embryonic kidney [76] and further transfilter culture experiments [77]. This basic finding shows that cell-to-cell communication exists and contradicts the general assumption that all of the involved morphogens are transported by diffusion. Actual morphological hints for a complex transport of morphogens are the spatial separation of mesenchymal and epithelial cell bodies in the niche and contacting mesenchymal cell projections (Figs. 2 and 3). Further, a striking interface filled with textured extracellular matrix and a basal lamina covering epithelial cells at the tip of a CD ampulla must be considered (Fig. 3b–d) [8, 48]. It is obvious that such an environment impedes diffusion and enables selective binding of morphogens during their transport on extracellular matrix [78]. In addition, contacts between mesenchymal and epithelial cells via cell projections including tunneling nanotubes makes it most probable that a route for a dosed transport of various molecules exists (Figs. 2, 4, and 5) [79].

**Fig. 6:**
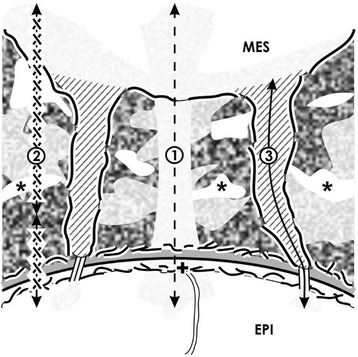
Schematic illustration informs about three possible routes for the transport of morphogens within the renal stem cell niche. Mesenchymal (MES) and epithelial (EPI) cells are separated by an interface (asterisk) including textured extracellular matrix. Projections of mesenchymal cells cross it to establish cell-to-cell contacts via tunneling nanotubes. On this situation, it is speculated that morphogens with good solubility are transported by diffusion (1). Morphogens with minor solubility are secreted in interstitial fluid and then bound on extracellular matrix, where they are delivered on demand (2). Morphogens with poor solubility are transported in cell projections and tunneling nanotubes (3). The basal lamina of epithelial stem cells at the tip of a CD ampulla is labeled by a cross

Finally, regarding not one but different kinds of transported morphogens, one has to consider that each of them has different molecular properties. They can be sorted according to good [19, 80, 81], minor [82, 83], and poor [84–86] solubility in saline solution [87]. Following this schema of sorting (Fig. 6), it is possible to allocate the transport of involved morphogens to recently detected morphological features (Figs. 2, 3, 4). Since concrete data for the human renal stem cell niche are not available, the suggested concept here is based on morphological findings raised in neonatal rabbit kidney and data collected from other developmental systems [88, 89].

### Ultrastructure restricts transport of morphogens

Fixation of specimens with glutaraldehyde (GA) solution including cupromeronic blue, ruthenium red, or tannic acid for transmission electron microscopy demonstrates textured extracellular matrix at the interface of the renal stem cell niche (Fig. 3b–d) [87, 88]. A complementary but only very little space does not show any label, appears to contain only interstitial fluid, and is consequently best suited for diffusion of molecules (Fig. 6.1). The sole candidate for a morphogen sprayed here is GDNF. The molecule contains 134 amino acids, it is secreted as a glycoprotein and it is well soluble in interstitial fluid [90]. According to literature, only GDNF synthesized by mesenchymal stem cells was up to date defined as a long-distance diffusible morphogen that binds on Ret tyrosine kinase receptor and a co-receptor GFRα1 localized at the tip of a CD ampulla [19, 91].

As mentioned, the available volume for long-distance diffusion of GDNF is unexpectedly small. In contrast, histochemical label of cupromeronic blue on mesenchymal cell projections and on the basal lamina covering epithelial stem/progenitor cells illustrates an unexpected amount of syndecans and/or glypicans (Fig. 3b), while label by ruthenium red (Fig. 3c) or tannic acid (Fig. 3d) points to perlecans and other proteins of extracellular matrix [92]. Recently published literature shows that especially proteoglycans exhibit a high affinity for morphogens and that they are able to modulate kidney development by interacting with GDNF, molecules of the FGF and TGFβ superfamilies, EGF receptor ligands, and HGF [93–96]. The present concept for the renal niche is that binding of individual morphogens on proteoglycans can be regarded as a “morphogenic switch” that triggers either as an inhibitor or as a facilitator the fine-tuning of a gradient and in turn the bioavailability of a morphogen (Fig. 6.2). This assumption is underlined by the fact that environment lacking heparan sulfate proteoglycans (HSPGs) does not support formation of an effective Wnt gradient abolishing in turn further development [92].

Other morphogens such as Wnt4, Wnt5a, and Wnt9b control renewal and differentiation of nephron progenitor cells, CD ampulla branching, and nephron induction [9, 97, 98]. One has to consider that Wnt molecules have posttranslational modifications in the form of a saturated palmitic acid and an unsaturated palmitoleic acid resulting in a poor solubility in saline impeding diffusion in interstitial fluid from one cell to the other [85]. There are hints that Wnt molecules are not secreted into interstitial fluid for further diffusion but are presented by epithelial cells near contacting mesenchymal cell projections. By this mechanism, Wnt molecules can immediately reach the plasma membrane of the target cell to find a directed cargo [99, 100]. Tkv-GFP receptor puncta of cell projections in *Drosophila* were shown to move here either in an anterograde or in a retrograde direction (Fig. 6.3) [101].

Morphogen sonic hedgehog (Shh) controls renal patterning [102, 103]. This kind of morphogen is not secreted into the interstitium but is distributed in the form of a particle that remains associated during transport with the surface on cell projections (Fig. 6.3) [104, 105].

Bone morphogenic proteins (BMPs) are morphogens with poor solubility in interstitial fluid [19]. For this reason, the transport of a BMP molecule from one cell to the other by diffusion in the interstitial fluid is unlikely. Instead, transport of BMPs at the contact site between a mesenchymal cell projection and an epithelial cell appears to be more probable (Fig. 6.3). At such cell contacts, released BMP molecules can bind on the plasma membrane for transport on receptors as it was demonstrated for *Drosophila* Tkv [106, 107].

### Tunneling nanotubes can direct controlled distribution

Actual morphological data exhibit that projections of mesenchymal stem cells cross the interface of a renal niche to penetrate the basal lamina and to contact the basal aspect of epithelia cells at the tip of a CD ampulla (Fig. 3a and 4) [87, 88]. In addition, between the end of a mesenchymal cell projection and the basal plasma membrane of an epithelial cell, tunneling nanotubes establish a basic functional cell-to-cell contact (Figs. 4 and 6.3). Occurrence of nanotubes at this specific site points out that in the niche, an up-to-date not considered bidirectional cargo system for organelles, membrane compounds, and other kinds of molecules exists [108–113].

Transport functions in the renal niche related to tunneling nanotubes were not under analysis up to date. Instead, other kinds of renal cells kept in culture were used to investigate cell-to-cell communication [55, 114]. Precise ligand distribution via cell-to-cell contacts and signaling filopodia was demonstrated for BMP2 and Wnt morphogens [115, 116]. Although yet not examined, it appears most likely that morphogens are transported in the renal niche this way, at the correct site and time, and in sufficient amount to maintain stemness and to initiate nephron induction (Fig. 6.3) [87, 88, 117].

### Export by vesicles

Beside described routes, it is also suspected that the transport of morphogens between cells takes place by vesicles such as exosomes (40–100 nm) or microvesicles (100–1000 nm) [118–120]. In such particles, principally as well mRNA, microRNA as synthesized morphogens can be shuttled [121–124]. Although demonstrated for regenerative processes in the kidney, with exception of morphogen Shh, literature is not available that informs about transport of morphogens by vesicles within the nephrogenic zone and contained niches [104, 105].

### Biomedicine encounters the nephrogenic zone

New literature exhibits that quite different influences can cause impairment of nephrogenesis in preterm and low-birth-weight babies [125]. It is obvious that independent from chemical nature, all of those hampering effects finally join the nephrogenic zone and affect the here contained niches. As described before, it is the definitive scene, where maintenance of stemness, precise positioning of cells, nephron induction, and initial development of nephrons are controlled. Consequently, therapeutic concepts prolonging nephrogenesis for preterm and low-birth-weight babies encounter inevitably the peculiar microanatomy of the nephrogenic zone, the complex interactions between nephrogenic mesenchymal and epithelial stem cells including the intact signaling of involved morphogens. In this situation, it is a consequent step to think about a therapeutic administration of morphogens. However, one must keep in mind that the here acting morphogens are on the one hand highly bio-effective molecules. On the other hand, one has to consider that a therapeutic use might be associated with hidden biomedical risks. For example, exogenously applied GDNF promotes formation of unwished ectopic ureteric buds [126].

Following notions for a therapeutic prolongation of nephrogenesis, the nephrogenic zone, contained niches, the bodies of mesenchymal and epithelial stem cells, their spatial separation by a striking interface, peculiar extracellular matrix, and cell-to-cell contacts via mesenchymal cell projections including tunneling nanotubes must be seen as a unique structural-functional ensemble (Figs. 2 and 3) [87, 88]. One has also to take into account that this area does not act autonomously but is under control of a set of morphogens with quite different biophysical features [9]. The actual problem is that about stimulation respectively inhibition of morphogen synthesis, secretion, and binding on extracellular matrix during local transport, only little concrete information is available. In other words, a site-specific therapy for the nephrogenic zone including the niches for prolongation of nephrogenesis in preterm infants needs first scientific answers to unsolved questions and then a critical selection of eligible drugs. Imaginable is either administration of a drug that stimulates the synthesis of the described genuine morphogens within the nephrogenic zone or administration of pharmacologically active morphogens by a smart drug delivery system.

### Safe drug delivery is the challenge

Due to an incomplete vascular supply, the nephrogenic zone is a bradytroph district (Fig. 5) [58–60]. At the site of stem cell niches, no intact vessels occur. This up-to-date less considered fact is a basic obstacle for a local drug administration prolonging nephrogenesis. Perhaps, it will be possible in the future to transport drugs via cortical radiate arteries and/or via the rete capsularis including the tunnel system [10]. In other words, first, it must be experimentally elaborated whether an administered drug shows a better distribution from the outer cortex or from the capsule side towards the nephrogenic zone.

In addition, morphogens such as GDNF or FGF8 with a good solubility in saline have a chance to reach niche sites by diffusion (Figure 6.1). For morphogens such as BMP4 or BMP7, a therapeutic administration will be more difficult, since secreted BMPs are transported by restricted diffusion and interaction with extracellular matrix (Fig. 6.2). Thus, in this case, not by the therapeutic administration of a drug but by the local extracellular matrix within the niche is decided upon free accessibility to the related receptor. For morphogens such as Wnt4, Wnt5a, Wnt9b, or Shh, an administration and action on the target cells becomes unpredictable. These morphogens are not soluble in saline solution and can bind optionally on extracellular matrix and on cell projections [74]. Transport of the mentioned morphogens is also thinkable along the plasma membrane and via tunneling nanotubes (Fig. 6.3) [127–129]. Possibly, isolation and culture of the nephrogenic zone in its original composition will help in a first issue to solve some of the mentioned problems [130].

## Conclusions

Preterm and low-birth-weight babies are suffering frequently on impaired nephrogenesis causing in turn oligonephropathy with lifelong disease risks. Consequently, a reliable pharmacological concept prolonging nephrogenesis is urgently required. However, due to incomplete vascular supply, complex microanatomy, and diverse signaling of morphogens, a site-specific drug administration in the nephrogenic zone will be a particular challenge. Thus, a drug may act, when it exhibits good solubility in interstitial fluid, to reach the target by long-distance diffusion. However, the effect of an administered drug is unpredictable, since it is most likely bound in the interstitial compartment on extracellular matrix. Depending on molecular structure and affinity, it is not constantly bioavailable but will be released by chance. However, administration, biophysical features, and bioavailability of a selected drug are not the sole problems. Its nephrotoxicity and possible side effects when acting in the nephrogenic zone and immature parenchyma must be analyzed even critically. Consequently, due to an unclear form of administration and unsure action of a drug on the related receptor, it is entirely unclear whether a therapeutic concept for prolongation of nephrogenesis will be available in the next future.
